# Trends in Filled Naloxone Prescriptions Before and During the COVID-19 Pandemic in the United States

**DOI:** 10.1001/jamahealthforum.2021.0393

**Published:** 2021-05-14

**Authors:** Ashley L. O’Donoghue, Nayantara Biswas, Tenzin Dechen, Timothy S. Anderson, Noa Talmor, Atulita Punnamaraju, Jennifer P. Stevens

**Affiliations:** 1 Center for Healthcare Delivery Science, Beth Israel Deaconess Medical Center, Boston, Massachusetts; 2 Department of Economics, Clark University, Worcester, Massachusetts; 3 Division of General Medicine, Beth Israel Deaconess Medical Center, Boston, Massachusetts; 4 Division for Pulmonary, Critical Care, and Sleep Medicine, Department of Medicine, Beth Israel Deaconess Medical Center, Boston, Massachusetts

## Abstract

This cohort study analyzes the trends in filled naloxone prescriptions during the COVID-19 pandemic in the United States and compare these to opioid prescriptions and overall prescriptions.

## Introduction

Substance use, including opioid use, increased during the COVID-19 pandemic.^1^ While overall emergency department visits decreased during the pandemic, nonfatal opioid overdose visits more than doubled, but few patients who overdosed on opioids received naloxone prescriptions on discharge.^2^ Studies show that increased access to naloxone can reduce fatal overdoses.^3,4^ In this study, we analyze the trends in filled naloxone prescriptions during the COVID-19 pandemic in the United States and compare these with trends in opioid prescriptions and overall prescriptions.

## Methods

Through the COVID-19 Research Database,^5^ we used Symphony Health, a pharmacy claims database that includes 92% of national retail pharmacy claims, 71% of mail-order pharmacy claims, and 65% of specialty pharmacy activity, to examine the period from May 2019 to December 2020. We analyzed weekly trends in the number of patients filling naloxone prescriptions, opioid prescriptions, and all prescriptions from May 13, 2019, to December 20, 2020. We defined the prepandemic time period to be May 13, 2019, through the week of March 13, 2020, when a national emergency was declared for COVID-19 in the United States. We defined the pandemic period as beginning the following week and continuing through the end of the sample period. We used an interrupted time series design to quantify changes in the level and growth rate in weekly filled prescriptions before and during the pandemic. We stratified analyses by payer (Medicaid, Medicare, commercial, and cash). We excluded weeks with national holidays (Thanksgiving, Christmas, and New Year’s Day). We used linear regression models and log-linear regression models, where a *P* value ≤.05 was considered statistically significant and all tests were 2-tailed. Stata SE version 16 (StataCorp) was used for statistical analysis. This research was classified as exempt by the Beth Israel Deaconess Medical Center institutional review board and it followed the Strengthening the Reporting of Observational Studies in Epidemiology (STROBE) reporting guideline for cohort studies. This study used only deidentified data and was classified as exempt from written informed consent by the Beth Israel Deaconess Medical Center institutional review board.

## Results

Trends in the average number of individuals filling prescriptions for naloxone, opioids, and any medication are summarized in the Figure. Abruptly in March 2020, the average number of individuals filling naloxone prescriptions per week declined in level by 361.09 individuals (95% CI, −499.56 to −222.62), which corresponds to a 26.32% reduction (Table). This exceeded the decline in the number of individuals filling prescriptions for any medication (−14.76%) and for opioid medications (−8.71%). Since March 2020, there was no statistically significant recovery in naloxone prescriptions (change in weekly growth rate: 0.36%; 95% CI, −0.17% to 0.88%), indicating that the number of individuals filling naloxone prescriptions has remained low throughout the pandemic. The weekly growth rates for individuals filling prescriptions for opioids and for any prescription have declined since March 2020. Individuals with Medicare and commercial insurance had a statistically significant decline in filling naloxone prescriptions at the start of the pandemic (−34.15% and −31.20%, respectively), while patients with Medicaid or cash payment had no statistically significant change during the pandemic.

**Figure.**
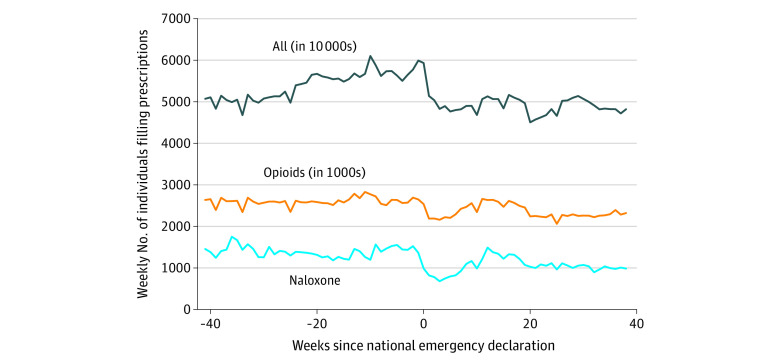
Trends in the Weekly Number of Individuals Filling Naloxone Prescriptions, Opioid Prescriptions, and All Prescriptions Trends in the weekly number of individuals filling naloxone prescriptions vs opioid prescriptions vs all prescriptions are from Symphony Health pharmacy claims data from May 13, 2019, to December 20, 2020. The *y* axis measures individuals filling naloxone prescriptions, individuals filling opioid prescriptions (measured in thousands), and individuals filling any prescriptions (measured in tens of thousands).

**Table.** Change in Level and Weekly Growth Rate of Naloxone, Opioid, and All Prescriptions From Prepandemic to During Pandemic^a^

Characteristic	Mean (95% CI)						
	Average No. of individuals (level)			% Change^d^	Weekly growth rate, % (trend)		
	Prepandemic level^b^	Pandemic level^b^	Change in level^c^		Prepandemic trend^e^	Pandemic trend^e^	Change in trend^f^
Individuals filling prescriptions							
Naloxone	1371.87 (1288.32 to 1455.42)^g^	1010.78 (897.09 to 1124.47)^g^	−361.09 (−499.56 to −222.62)^g^	−26.32	−0.07 (−0.31 to 0.19)	0.29 (−0.20 to 0.78)	0.36 (−0.17 to 0.88)
Opioids (in thousands)	2651.46 (2590.49 to 2712.42)^g^	2420.49 (2322.72 to 2518.26)^g^	−230.97 (−343.93 to −118.01)^g^	−8.71	0.09 (−0.01 to 0.18)	−0.15 (−0.33 to 0.04)	−0.23 (−0.43 to −0.03)^g^
All (in tens of thousands)	5915.71 (5809.23 to 6022.19)^g^	5042.76 (4895.40 to 5190.11)^g^	−872.95 (−1051.36 to −694.54)^g^	−14.79	0.46 (0.37 to 0.54)^g^	−0.11 (−0.25 to 0.02)	−0.57 (−0.72 to −0.42)^g^
Individuals filling naloxone by payer							
Medicaid	184.10 (169.46 to 198.74)^g^	180.94 (163.49 to 198.38)^g^	−3.16 (−25.54 to 19.22)	−1.72	−0.55 (−0.82 to −0.29)^g^	0.05 (−0.40 to 0.50)	0.60 (0.10 to 1.10)^g^
Medicare	552.58 (510.28 to 596.88)^g^	364.90 (317.34 to 412.46)^g^	−188.68 (−251.91 to −125.45)^g^	−34.15	−0.01 (−0.33 to 0.31)	0.51 (−0.05 to 1.07)	0.52 (−0.10 to 1.15)
Commercial	527.93 (497.98 to 557.88)^g^	363.19 (318.79 to 407.59)^g^	−164.74 (−217.27 to −112.21)^g^	−31.20	0.04 (−0.20 to 0.27)	0.44 (−0.07 to 0.96)	0.41 (−0.14 to 0.95)
Cash	106.26 (96.17 to 116.36)^g^	101.75 (91.03 to 112.47)^g^	−4.51 (−18.99 to 9.97)	−4.24	0.11 (−0.30 to 0.51)	−0.73 (−1.26 to −0.21)^g^	−0.84 (−1.49 to −0.19)^g^

^a^ The prepandemic period is defined as May 13, 2019, through the week of March 13, 2020. The pandemic period begins the following week and continues through December 20, 2020.

^b^ Predicted levels are calculated from a linear regression of weekly trends on the number of individuals filling prescriptions.

^c^ The change in level is calculated as the coefficient on the term that measures the breakpoint in the interrupted time series analysis.

^d^ Calculated as (change in level/prepandemic level × 100).

^e^ The trend is calculated as the coefficient on the weekly trend in a log-linear regression, where the dependent variable (number of individuals filling a prescription) is log transformed.

^f^ The change in trend is calculated as the coefficient on the interaction term in the interrupted time series analysis where the dependent variable (number of individuals filling a prescription) is log transformed.

^g^ Denotes *P* ≤ .05.

## Discussion

These findings indicate that individuals on Medicare and commercial insurance may be experiencing decreased access to naloxone during the COVID-19 pandemic. The decline is not explained by a decline in opioid prescriptions, where naloxone coprescription is recommended, as opioid prescriptions only decreased by 8.71% in March 2020, less than all prescriptions and much less than naloxone prescriptions. Limitations of this study include that we are only able to account for naloxone prescriptions filled at retail pharmacies and not naloxone kits distributed from other sources, such as syringe service programs.^6^ Our study identifies an urgent gap in necessary access to medication for individuals on Medicare and commercial insurance during the pandemic. Continuing to increase naloxone distribution in densely populated areas and via mail order and delivery through community-based organizations could help to mitigate some of the reductions in naloxone distribution via pharmacies and could reduce some of the increases in fatal opioid overdoses during the COVID-19 pandemic.
